# Clinical significance of focal ß-amyloid deposition measured by ^18^F-flutemetamol PET

**DOI:** 10.1186/s13195-019-0577-x

**Published:** 2020-01-04

**Authors:** Si Eun Kim, Byungju Lee, Seongbeom Park, Soo Hyun Cho, Seung Joo Kim, Yeshin Kim, Hyemin Jang, Jee Hyang Jeong, Soo Jin Yoon, Kyung Won Park, Eun-Joo Kim, Na Yeon Jung, Bora Yoon, Jae-Won Jang, Jin Yong Hong, Jihye Hwang, Duk L. Na, Sang Won Seo, Seong Hye Choi, Hee Jin Kim

**Affiliations:** 1 Department of Neurology, Samsung Medical Center, Sungkyunkwan University School of Medicine, 50 Ilwon-dong, Gangnam-ku, Seoul, 135-710 Republic of Korea; 2 Department of Neurology, Inje University College of Medicine, Haeundae Paik Hospital, Busan, Korea; 3 Department of Neurology, Yuseong Geriatric Rehabilitation Hospital, Pohang, Korea; 4 Samsung Alzheimer Research Center, Samsung Medical Center, Seoul, Korea; 5 Neuroscience Center, Samsung Medical Center, Seoul, Korea; 6 Department of Neurology, Chonnam National University Hospital, Chonnam National University Medical School, Gwangju, Korea; 7 Department of Neurology, Gyeongsang National University School of Medicine and Gyeongsang National University Changwon Hospital, Changwon, Korea; 8 Department of Neurology, Kangwon National University Hospital, Kangwon National University College of Medicine, Chuncheon, Korea; 9 Department of Neurology, Ewha Womans University Mokdong Hospital, Ewha Womans University School of Medicine, Seoul, Korea; 10 Department of Neurology, Eulji University Hospital, Eulji University School of Medicine, Daejeon, Korea; 11 Department of Neurology, Dong-A Medical Center, Dong-A University College of Medicine, Busan, Korea; 12 Department of Neurology, Pusan National University Hospital, Pusan National University School of Medicine and Medical Research Institute, Busan, Korea; 13 Department of Neurology, Pusan National University Yangsan Hospital, Pusan National University School of Medicine and Research Institute for Convergence of Biomedical Science and Technology, Yangsan, Korea; 14 Department of Neurology, Konyang University College of Medicine, Daejeon, Korea; 15 Department of Neurology, Yonsei University Wonju College of Medicine, Wonju, Korea; 16 Department of Neurology, Keimyung University Daegu Dongsan Hospital, Daegu, Korea; 17 Department of Health Sciences and Technology, SAIHST, Sungkyunkwan University, Seoul, Korea; 18 Stem Cell & Regenerative Medicine Institute, Samsung Medical Center, Seoul, Korea; 19 Department of Clinical Research Design and Evaluation, SAIHST, Sungkyunkwan University, Seoul, Korea; 20 Center for Clinical Epidemiology, Samsung Medical Center, Seoul, Korea; 21 Department of Neurology, Inha University School of Medicine, Incheon, Korea

**Keywords:** ß-amyloid, ^18^F-flutemetamol PET, Alzheimer’s disease, Cognition, Cerebrospinal fluid

## Abstract

**Background:**

Although amyloid PET of typical Alzheimer’s disease (AD) shows diffuse ß-amyloid (Aß) deposition, some patients show focal deposition. The clinical significance of this focal Aß is not well understood. We examined the clinical significance of focal Aß deposition in terms of cognition as well as Aß and tau cerebrospinal fluid (CSF) levels. We further evaluated the order of Aß accumulation by visual assessment.

**Methods:**

We included 310 subjects (125 cognitively unimpaired, 125 mild cognitive impairment, and 60 AD dementia) from 9 referral centers. All patients underwent neuropsychological tests and ^18^F-flutemetamol (FMM) PET. Seventy-seven patients underwent CSF analysis. Each FMM scan was visually assessed in 10 regions (frontal, precuneus and posterior cingulate, lateral temporal, parietal, and striatum of each hemisphere) and was classified into three groups: No-FMM, Focal-FMM (FMM uptake in 1–9 regions), and Diffuse-FMM (FMM uptake in all 10 regions).

**Results:**

53/310 (17.1%) subjects were classified as Focal-FMM. The cognitive level of the Focal-FMM group was better than that of Diffuse-FMM group and worse than that of No-FMM group. Among the Focal-FMM group, those who had FMM uptake to a larger extent or in the striatum had worse cognitive levels. Compared to the Diffuse-FMM group, the Focal-FMM group had a less AD-like CSF profile (increased Aß42 and decreased t-tau, t-tau/Aß42). Among the Focal-FMM group, Aß deposition was most frequently observed in the frontal (62.3%) and least frequently observed in the striatum (43.4%) and temporal (39.6%) regions.

**Conclusions:**

We suggest that focal Aß deposition is an intermediate stage between no Aß and diffuse Aß deposition. Furthermore, among patients with focal Aß deposition, those who have Aß to a larger extent and striatal involvement show clinical features close to diffuse Aß deposition. Further longitudinal studies are needed to evaluate the disease progression of patients with focal Aß deposition.

**Electronic supplementary material:**

The online version of this article (10.1186/s13195-019-0577-x) contains supplementary material, which is available to authorized users.

## Background

Alzheimer’s disease (AD), the most common cause of dementia, is characterized by ß-amyloid (Aß) and tau accumulation in the brain [1]. Aß accumulation starts approximately 10 to 20 years before dementia symptoms begin. Thus, detecting the presence of Aß is essential for early diagnosis of AD. With recent advances in detecting Aß in vivo, the use of an Aß biomarker is clinically available through PET imaging or CSF analysis [2, 3].

Although amyloid PET of typical AD shows diffuse Aß deposition, some patients show focal Aß deposition, the clinical significance of which is not well defined. Previous studies lack pathological examination results of patients with focal Aß deposition on PET imaging. Brain Aß burden on PET imaging may be quantitatively measured in the research field using standardized uptake value ratios (SUVR) and studies showed that higher SUVR is correlated with poor clinical outcome [4–6]. Meanwhile, in clinical practice, interpretation of amyloid PET relies on dichotomous visual rating (positive or negative). According to visual interpretation guidelines of PET images such as ^18^F-florbetaben or ^18^F-flutemetamol (FMM), if any of the brain regions (frontal, parietal, precuneus/posterior cingulate (PPC), lateral temporal lobes, and striatum) is positive in either hemisphere, the scan is considered to be positive [7, 8]. However, since Aß deposition is a gradual process [9], a dichotomous visual rating may be misleading. Identifying the clinical significance of participants in the gray zone may help manage patients in clinical practice. Thus, this particular group needs to be well characterized.

In this study, we hypothesized that patients showing focal Aß deposition have unique clinical characteristics. We examined patients showing focal Aß deposition on FMM PET scan. We compared cognition and CSF AD biomarkers between patients with No-, Focal-, and Diffuse-FMM uptake. We also aimed to assess whether the extent and region of focal FMM uptake are related to cognition. We further evaluated the order of Aß accumulation by visual assessment.

## Methods

### Participants

We recruited 310 patients with cognitively unimpaired (CU; *n* = 125), mild cognitive impairment (MCI; *n* = 125), and AD dementia (ADD, *n* = 60) who underwent FMM PET between June 2015 and December 2017. The CU individuals had normal age-, sex-, and education-adjusted performance on standardized cognitive tests [10]. The participants with MCI met the criteria proposed by Petersen et al. [11]: (1) subjective memory complaints, (2) relatively normal performance in other cognitive domains, (3) normal activities of daily living (ADL), (4) objective memory impairment below − 1.5 SD on either verbal or visual memory tests, and (5) not demented. The ADD patients met the criteria for dementia by the Diagnostic and Statistical Manual of Mental Disorders 4th Edition, Text Revision (DSM-IV-TR) [12] and were diagnosed with probable ADD according to the NIA-AA core clinical criteria [1]. The patients were recruited from 9 referral hospitals in South Korea (175 from Samsung Medical Center, 135 from Validation Cohort of Korean Brain Aging Study for the Early Diagnosis and Prediction of AD (KBASE-V) [13]). All participants underwent neurologic examination, neuropsychological test, and Apolipoprotein E (*APOE*) genotyping. We screened blood tests including a complete blood count, blood chemistry, thyroid function, vitamin B12, folate, and syphilis serology and excluded participants with abnormal findings that could affect their cognition. Participants with previous or current neurological or psychiatric diseases such as brain tumors, encephalitis, epilepsy, and depressive disorders that could affect cognitive function were also excluded. On MRI, patients with structural lesions such as hydrocephalus, brain tumors, or traumatic brain injuries were also excluded. The Institutional Review Boards approved this study at all participating centers. We obtained written, informed consent from patients and caregivers.

### ^18^F-flutemetamol PET acquisition and analysis

We performed FMM PET scans using a Discovery 600 PET/CT scanner (GE), Discovery 690 PET/CT scanner (GE), Discovery STE PET/CT scanner (GE), Biography MCT PET/CT scanner (Siemens) or Gemini TF PET/CT scanner (Philips) on a total number of 310 participants as described in a previous study [13]. The participants underwent a 20 min PET scan (4 × 5 min dynamic frames) starting at 90 min after intravenous injection of 185 MBq ± 10% of ^18^F-flutemetamol. Low-dose computed tomography was utilized for attenuation correction before scans. We reconstructed the images with the Ordered Subsets Expectation Maximization algorithm using 4 iterations and 16 subsets.

### Blinded visual interpretation

Visual interpretation of the FMM PET images was performed by two blinded neurologists (referred to as “readers”) who successfully completed the manufacturing company’s electronic training program. Visual interpretation of FMM PET images was performed by systematic review of ten brain regions (frontal, parietal, PPC, striatum, and lateral temporal lobes in each hemisphere) [14]. For each region, the readers used dichotomous assessment in classifying images as either normal or abnormal in a rainbow color scale anchored to the pons. We defined each region to be abnormal when there was increased cortical gray matter signal (above 50–60% peak intensity) and/or reduced (or absent) gray-white matter contrast [15]. Inter-reader agreement of interpretation of FMM PET was excellent (kappa score = 0.94).

We classified patients into three groups. No-FMM (no FMM uptake in any region), Focal-FMM (FMM uptake in 1–9 regions), and Diffuse-FMM (FMM uptake in all 10 regions). Examples of FMM PET images are shown in Additional file 1: Figure S1.

### Cerebrospinal fluid collection, processing, and analysis

CSF sampling was performed in 77 participants (49 CU, 16 MCI, and 12 ADD) by procedures as previously described [13]. We obtained CSF in 15 mL polypropylene tubes (Falcon, Corning Science, NY, USA) and centrifuged at 2000×*g* for 10 min at room temperature (RT). Approximately 10 cc of the CSF supernatant was frozen and transferred to the laboratory at Inha University. For measuring CSF biomarkers, the CSF was thawed and gently extracted into pipettes with polypropylene tips. A total of 0.4 mL aliquots of CSF was frozen in polypropylene tubes (Sarstedt AG & Co., Nümbrecht, Germany) and stored at − 80 °C until analysis. We measured the level of CSF Aß 42, total tau (t-tau), and phosphorylated tau (p-tau) using the multiplex xMAP Luminex platform with INNO-BIA AlzBio3 kits. AlzBio3 kits (Fujirebio Europe, Ghent, Belgium) contained capture monoclonal antibodies for Aß 42, t-tau, and p-tau, which linked to two aqueous quality controls (a-QC) with pre-defined concentration ranges for the three biomarkers. The procedure is described elsewhere [16, 17]. To reduce the effects of sources of variability on the results [18], CSF analysis was followed by the manufacturer’s instructions and standard of procedures that were previously described [19, 20].

### Neuropsychological evaluation

All participants underwent a standardized neuropsychological battery called the Korean version of the Consortium to Establish a Registry for Alzheimer’s Disease Assessment Packet (CERAD-K) [21] or Seoul Neuropsychological Screening Battery (SNSB) [22], which consisted of tests of language, visuospatial, memory, and frontal/executive functions.

Tests in CERAD-K included the Korean version of the Boston Naming Test (K-BNT) to assess language function; constructional praxis (copying figures) to assess visuospatial function; 10 word list recall (20-min delayed recall) and constructional recall (20-min delayed figure recall) to assess verbal and visual memory, respectively; Controlled Oral Word Association Test (COWAT: animal naming) and Stroop Test (color reading) to assess frontal/executive function; and the Mini-Mental State Examination (MMSE) to assess global cognitive function.

Tests in SNSB included the K-BNT to assess language function; Rey-Osterrieth Complex Figure Test (RCFT: copying) to assess visuospatial function; Seoul Verbal Learning Test (20-min delayed recall) and RCFT (20-min delayed recall) to assess verbal and visual memory, respectively; COWAT (animal naming) and the Stroop Test (color reading) to assess frontal-executive function; and the MMSE to assess global cognitive function. [23]

Tests were conducted by experienced staff and supervised by board-certified neuropsychologists. The norms for each test were based on 1987 normal Korean participants (for CERAD-K) or 1067 normal Korean participants (for SNSB). In the analyses, we used the *z*-scores of each test, which were based on the mean and standard deviation of each measure in the age- and education-matched norms.

### Statistical analysis

For descriptive statistics, we used the chi-square test and analysis of variance (ANOVA) followed by Bonferroni’s post hoc analyses.

To compare the cognitive profile of the three groups (No-FMM, Focal-FMM, and Diffuse-FMM group), we used ANOVA followed by Bonferroni’s post hoc analyses. When we compared the cognitive profile of the Regional-FMM group with the No-FMM or the Diffuse-FMM group, we used ANOVA followed by Bonferroni’s correction for 30 multiple tests (5 regions and 6 cognitive tests). To evaluate the association between cognition and number of FMM uptake regions, we used linear regression analyses.

For comparison of the CSF profile of the three groups, we used analysis of covariance (ANCOVA) followed by Bonferroni’s post hoc analyses after controlling for age. To evaluate the association between CSF profile and number of FMM uptake regions, linear regression analyses were used after adjusting for age. All statistical tests were performed using MedCalc (MedCalc Software version 19, Ostend, Belgium).

To determine the spreading order of FMM uptake, we assumed that regions with earlier appearance of pathology would show abnormal FMM uptake in a greater number of participants, as suggested by previous studies [24, 25]. The different frequency of regional involvement was assessed using a bootstrapping method with 1000 re-samples in R v3.1.3 (Institute for Statistics and Mathematics, Vienna, Austria; www.R-project.org), which derived the estimates of 95% confidence intervals and standard error. We calculated asymptotic *p* values and corrected for multiple comparisons with Bonferroni’s method for all combinations of regional pairs.

## Results

### Characteristics of the participants

The demographic and clinical characteristics are presented in Table 1. Of all the participants, 17.1% (53/310) patients were classified into the Focal-FMM group. The proportion in the Focal-FMM group and the extent of Focal-FMM uptake differed according to cognitive level. 13.6% (17/125) of CU, 16.0% (20/125) of MCI, and 26.7% (16/60) of ADD were classified into the Focal-FMM group. Among the Focal-FMM group, the median number of uptake regions was 1.0 (95% CI = 1.0–4.0) in CU, 3.5 (95% CI = 2.0–5.8) in MCI, and 6.0 (95% CI = 3.6–8.0) in ADD. The Focal-FMM group was older than the No-FMM group and had more *APOE ε4* carriers compared to the No-FMM group. In addition, there were statistically significant differences in distribution of cognitive level across the groups.

**Table 1:** Demographic characteristics of participants

	No-FMM uptake (*n* = 174)	Focal-FMM uptake (*n* = 53)	Diffuse-FMM uptake (*n* = 83)	*p* No vs focal uptake	*p* No vs diffuse uptake	*p* Focal vs diffuse uptake
Age (mean ± SD)	69.4 ± 8.6	73.5 ± 7.9	71.4 ± 8.5	0.008	0.253	0.494
Men (%)	73 (42.0)	15 (28.3)	37 (44.6)	0.075	0.692	0.058
Education, years (mean ± SD)	10.7 ± 5.0	10.3 ± 5.0	11.0 ± 4.5	1.000	1.000	1.000
*APOE* ε*4* carrier (%)	21/166 (12.7)	23/53 (43.4)	42/81 (51.9)	< 0.001	< 0.001	0.340
Disease duration (months) (Mean ± SD)	62.5 ± 52.9	50.3 ± 44.5	41.5 ± 41.6	0.455	0.009	1.000
Vascular risk factors						
Hypertension (%)	80 (46.0)	23 (43.4)	29 (34.9)	0.742	0.095	0.324
Diabetes (%)	33 (19.0)	5 (9.4)	11 (13.3)	0.104	0.257	0.502
Hyperlipidemia (%)	57 (32.8)	18 (34.0)	11 (13.3)	0.871	0.001	0.004
Cognitive level				< 0.001	< 0.001	< 0.001
CU (%)	102 (58.6)	17 (32.1)	6 (7.2)			
MCI (%)	64 (36.8)	20 (37.7)	41 (49.4)			
ADD (%)	8 (4.6)	16 (30.2)	36 (43.4)			

*Abbreviations*: *FMM* ^18^F-flutemetamol, *APOE* ε*4* Apolipoprotein ε*4*, *CU* cognitively unimpaired, *MCI* mild cognitive impairment, *ADD* Alzheimer’s disease dementia

### Cognitive profiles of Focal-FMM group

Compared to the No-FMM group, the Focal-FMM group showed significantly lower performance in all cognitive domains except for visuospatial function. Compared to the Diffuse-FMM group, the Focal-FMM group showed better performance in verbal memory and visual memory functions. Global cognitive function (measured by MMSE) of the Focal-FMM group was better than the Diffuse-uptake group but worse than the No-FMM group (Table 2).

**Table 2:** Cognitive profile according to ^18^F-flutemetamol uptake regions

Neuropsychological tests of cognitive domain	No-FMM uptake	Focal-FMM uptake						Diffuse-FMM uptake	No vs focal uptake	No vs diffuse uptake	Focal vs diffuse uptake
	(*n* = 174)	Total (*n* = 53)	Frontal (*n* = 33)	Lateral temporal (*n* = 21)	Parietal (*n* = 32)	PPC (*n* = 32)	Striatum (*n* = 23)	(*n* = 83)			
Language											
K-BNT	0.02 ± 1.17	− 0.94 ± 1.78	− 1.33 ± 1.68*	− 1.27 ± 1.93	−1.40 ± 2.00*	−1.50 ± 1.97*	−1.80 ± 2.00*	− 0.97 ± 1.50	< 0.001	< 0.001	1.000
Visuospatial											
Figure copying	− 0.12 ± 1.31	− 0.71 ± 2.19	− 1.10 ± 2.54	− 0.55 ± 2.02	−0.95 ± 2.55	− 1.37 ± 2.53	−1.53 ± 2.82	− 1.62 ± 3.76	0.324	< 0.001	0.089
Memory											
Verbal memory	− 0.46 ± 1.26	− 0.97 ± 1.26	− 1.12 ± 1.19^#^	− 0.97 ± 1.31^#^	− 1.05 ± 1.40^#^	− 1.32 ± 1.27*^#^	−1.63 ± 0.84*	−2.12 ± 1.04	0.020	< 0.001	< 0.001
Visual memory	− 0.18 ± 1.10	− 0.83 ± 1.15	− 1.08 ± 1.06*^#^	− 0.68 ± 1.05^#^	− 0.93 ± 1.19*^#^	− 1.09 ± 1.21*	− 1.34 ± 1.08*	−1.77 ± 0.96	< 0.001	< 0.001	< 0.001
Frontal/executive											
COWAT	− 0.07 ± 1.10	− 0.78 ± 1.11	− 0.98 ± 1.08*	− 1.09 ± 1.02*	− 0.89 ± 1.30*	− 1.17 ± 1.04*	−1.32 ± 1.02*	− 1.09 ± 1.11	< 0.001	< 0.001	0.331
Stroop test	− 0.06 ± 1.42	− 0.60 ± 1.63	− 0.93 ± 1.67	− 0.66 ± 1.57	− 0.91 ± 1.81	− 1.18 ± 1.66*	− 1.48 ± 1.54*	− 1.66 ± 1.77	0.080	< 0.001	< 0.001
Global											
MMSE	− 0.31 ± 1.35	− 1.13 ± 2.46	− 1.65 ± 2.80	− 1.04 ± 1.86	− 1.35 ± 2.86	− 1.85 ± 2.84	−2.24 ± 3.21	− 2.57 ± 2.98	0.039	< 0.001	< 0.001
CDR-SB	0.80 ± 1.04	2.19 ± 2.63	2.94 ± 2.82*	2.55 ± 2.43	2.50 ± 2.87	3.17 ± 2.90*	3.52 ± 3.18*	3.10 ± 2.68	< 0.001	< 0.001	0.024

Values are mean *z*-scores of neuropsychological tests and raw score of CDR-SB (mean ± SD)

*p < 0.05 between regional Focal-FMM-uptake group and No-FMM-uptake group (after Bonferroni’s correction for 5 regions and 6 cognitive tests)

#*p* < 0.05 between regional Focal-FMM-uptake group and Diffuse-FMM-uptake group (after Bonferroni’s correction for 5 regions and 6 cognitive tests)

*Abbreviations*: *FMM* ^18^F-flutemetamol, *COWAT* The Controlled Oral Word Association Test, *K-BNT* Korean version-Boston Naming Test, *MMSE* Mini-Mental State Exam, *CDR-SB* Clinical Dementia Rating Scale Sum of Boxes

**Fig. 1:**
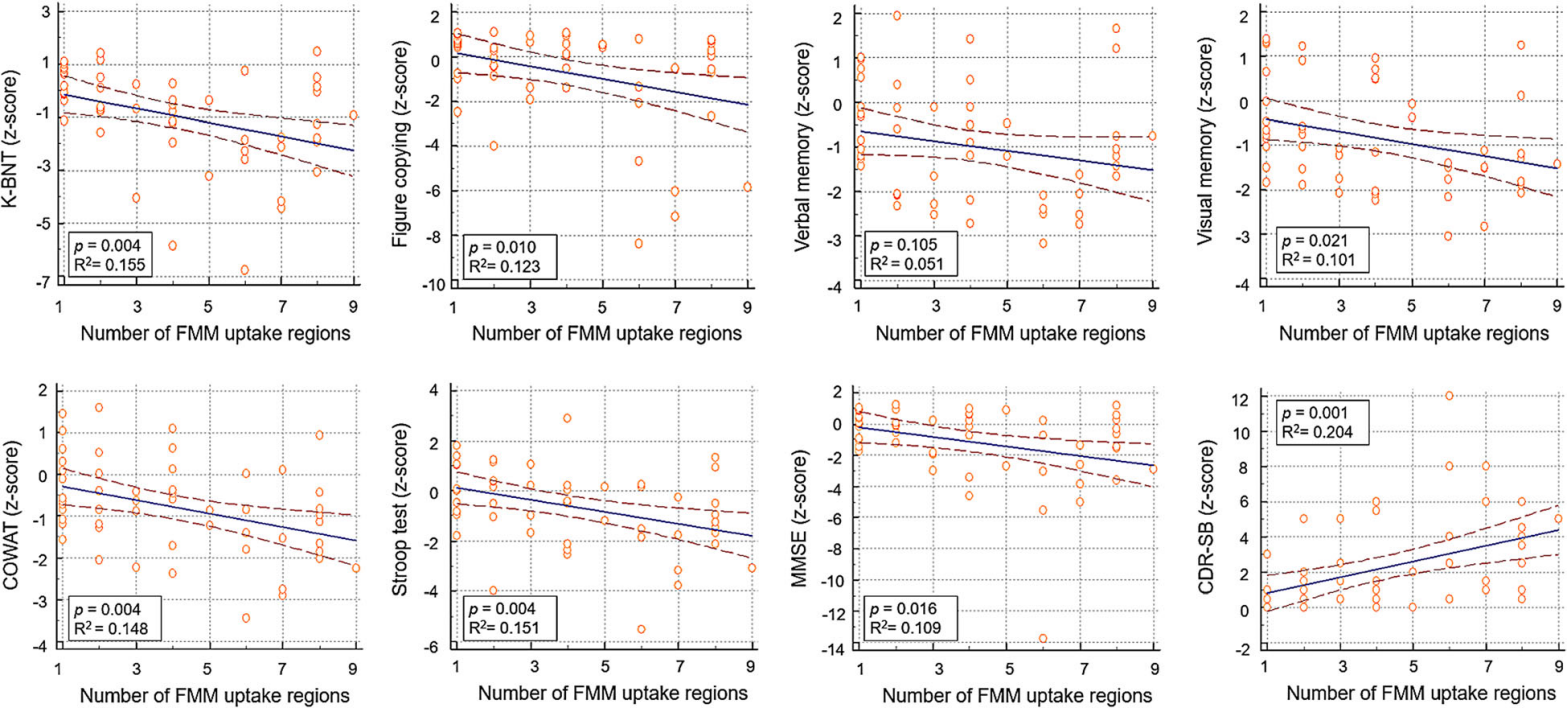
Cognitive profile according to number of FMM uptake regions among the Focal-FMM group. Solid blue line is the regression line. Brown dotted lines indicate 95% confidence intervals. Abbreviations: FMM = ^18^F-flutemetamol; K-BNT = Korean version-Boston Naming Test; COWAT = The Controlled Oral Word Association Test; MMSE = Mini-Mental State Exam

Among the Focal-FMM group, as the number of FMM uptake regions increased, *z*-scores decreased in all cognitive domains such as K-BNT (β = − 0.264, *p* = 0.004), visuospatial function (*β* = − 0.290, *p* = 0.010), verbal memory (*β* = − 0.105, *p* = − 0.105), visual memory (*β* = − 0.138, *p* = 0.021), COWAT (*β* = − 0.162, *p* = 0.004), Stroop Test (*β* = − 0.239, *p* = 0.004), and MMSE (*β* = − 0.306, *p* = 0.016) **(**Fig. 1**.)**.

We further divided Focal-FMM group into patients with less FMM uptake (1–5 regions involved) group and patients with more FMM uptake (6–9 regions involved) group. Focal-FMM group with 1–5 regions involved did not show significant difference compared to the No-FMM group, while it showed better performance in all cognitive domains except for language when compared to Diffuse-FMM group. Focal-FMM group with 6–9 regions involved showed worse performance in all cognitive domains compared to No-FMM group, while it did not show significant difference when compared to Diffuse-FMM group (Additional file 1: Table S1).

Then, we compared each regional Focal-FMM group with the Focal- or Diffuse-FMM group to evaluate the regional effects of focal FMM uptake on cognitive function. Compared to the Diffuse-FMM group, verbal memory scores were higher in the Focal-FMM group with frontal, lateral temporal, parietal, or PPC regional involvement whereas no difference was found in the Focal-FMM group with striatal involvement. Compared to the Diffuse-FMM group, visual memory scores were higher in the Focal-FMM group with frontal, lateral temporal, or parietal regional involvement whereas no difference was found in the Focal-FMM group with PPC or striatal involvement. Compared to the No-FMM group, verbal and visual memory scores were lower in the Focal-FMM group with PPC or striatal involvement (Table 2).

Comparisons of cognitive scores between the No-, Focal-, and Diffuse-FMM groups in each cognitive level (CU, MCI and ADD) are shown in Additional file 1: Table S2). Among CU individuals, the cognitive score did not differ among the No-, Focal-, and Diffuse-FMM groups. However, among MCI patients, the Focal-FMM group showed better performance in verbal and visual memory function, as well as in MMSE score, compared to the Diffuse-FMM group. Among ADD patients, Focal-FMM patients performed worse in language and frontal function compared to those of the No-FMM group and performed better in verbal memory than those of the Diffuse-FMM group. We provided a breakdown of cases by clinical designation and number of regions in Additional file 1: Table S3).

### CSF amyloid and tau level of focal-FMM group

**Fig. 2:**
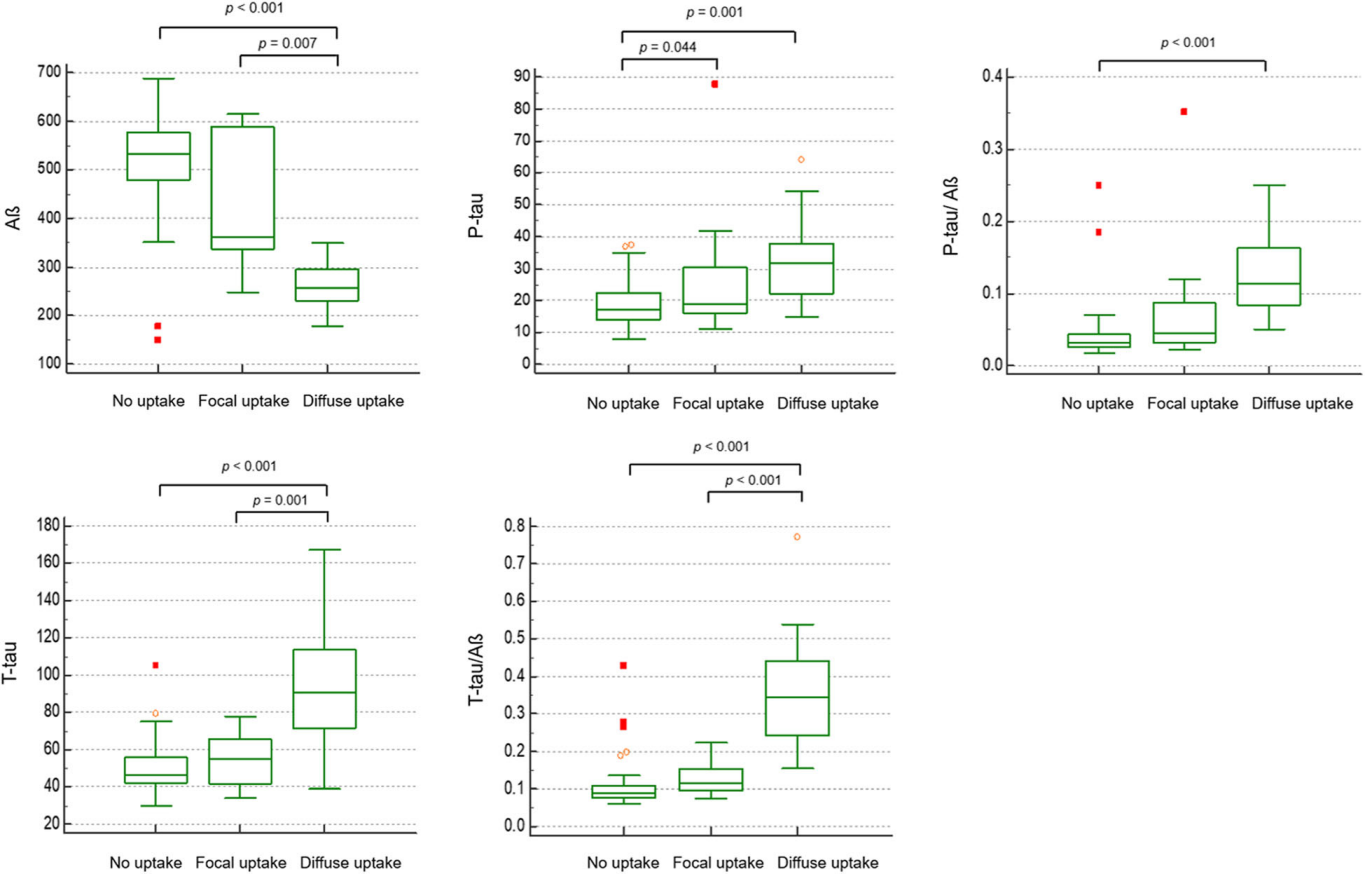
Comparison of Alzheimer’s disease biomarkers (Aß, P-tau, and T-tau) from cerebrospinal fluid among No-, Focal-, and Diffuse-FMM groups after adjusted for age. Box and whisker plots show medians, lower to upper quartile, and lines extending from minimum to maximum values. Abbreviations: FMM = ^18^F-flutemetamol; P-tau = phosphorylated tau; T-tau = total tau

Levels of CSF AD biomarkers (Aß 42, p-tau, t-tau, p-tau/ Aß 42, and t-tau/ Aß 42) in No-, Focal-, and Diffuse-FMM groups are shown in Fig. 2. The Focal-FMM group showed increased levels of CSF Aß 42 and decreased levels of CSF t-tau and t-tau/Aß 42, compared to the Diffuse-FMM group. However, there were no differences between Focal-FMM-uptake and No-FMM-uptake groups except for p-tau level.

### Spreading order of FMM-uptake among focal-FMM-uptake group

**Fig. 3:**
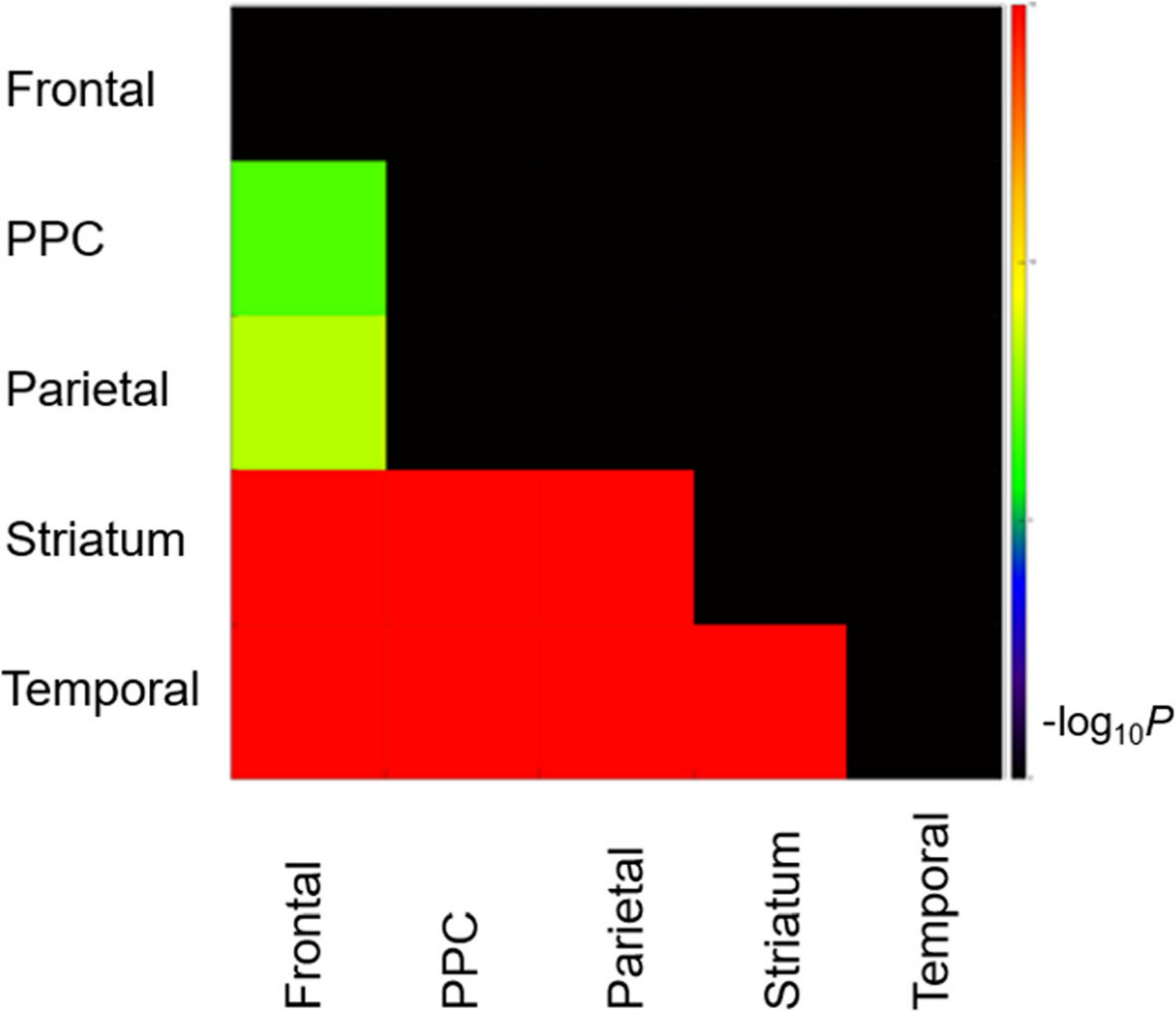
Spreading order of FMM among Focal-FMM group (*N* = 53). It shows the statistical significance in the comparison of the frequencies of FMM regional involvement between each pair of regions. The regional differences of uptake frequency display a stepwise pattern. Only the pairs of comparison passing Bonferroni’s multiple comparisons are shown. Color bars represent logarithmic scale of *p* value (−log_10_). Abbreviation: PPC = precuneus/posterior cingulate

Among the Focal-FMM group, Aß deposition was most frequently observed in the frontal (62.3%, 95% CI = 48.8–75.8) followed by PPC (60.4%, 95% CI = 46.8–74.0), parietal (60.4%, 95% CI = 46.8–74.0), striatum (43.4%, 95% CI = 29.6–57.2), and lateral temporal (39.6%, 95% CI = 26.0–53.2) regions (Fig. 3).

## Discussion

In this study, we investigated the clinical significance of patients with Focal-FMM uptake, which consisted of 17.1% of all participants. Our major findings were as follow. First, cognitive function of patients with Focal-FMM uptake was in the intermediate stage between patients with No- and Diffuse-FMM uptake. Among Focal-FMM patients, the larger extent and striatal involvement of FMM uptake was associated with worse cognition. Second, CSF AD biomarkers of Focal-FMM group were less AD-like compared to the Diffuse-FMM group. Finally, among the Focal-FMM group, FMM uptake was most frequently observed in the frontal and least observed in the striatum and lateral temporal regions. Taken together, our findings suggest that patients with Focal-FMM uptake have unique clinical characteristics. Furthermore, among patients with focal FMM uptake, those who have larger extent and striatal involvement of FMM showed clinical features close to diffuse Aß uptake and, thus, might be considered as being in more advanced stage of AD.

We found that 17.1% of all participants had Focal-FMM uptake. Focal-FMM consisted substantial portion of participants in each cognitive level: 13.6% (17/125) of UC, 16.0% (20/125) of MCI, and 26.7% (16/60) of ADD. In clinical practice, interpretation of amyloid PET relies on visual assessment, which currently guides focal-FMM uptake to be read as positive for Aß. However, the clinical significance of focal Aß uptake is not well understood. Characterizing participants in this gray zone may help better manage patients.

Our first major finding was that cognitive function of patients with Focal-FMM was in the intermediate stage between patients with No- and Diffuse-FMM uptake. More importantly, the cognitive function differed according to the number and location of regions of focal FMM uptake. Among the Focal-FMM group, cognitive scores decreased with increasing number of FMM uptake regions. Our results are in line with previous studies using quantitative measurement of Aß burden [PIB [26–28], ^18^F-florbetapir [29], or FMM [30] SUVR]. Subjects with higher PiB SUVR showed lower scores on episodic memory tests [27]. Higher FMM SUVR correlated with lower delayed memory index [30], and higher ^18^F-florbetapir SUVR correlated with lower MMSE scores [29]. Among the Focal-FMM group, those who had FMM uptake in the striatum had the worst cognitive scores. Although there have been numerous studies on the associations between quantitative Aß deposition and cognition, no study has reported the association between visually assessed Aß deposition and cognitive profiles. As quantitative analysis is not widely used in clinical practice, studies on visual assessment is valuable for clinicians. Our results suggest that when managing patients showing focal FMM uptake, clinicians should consider the number and location of regions with focal Aß deposition.

Our second major finding was that patients with focal Aß deposition on PET showed less AD-like CSF profiles compared to the Diffuse-FMM group. In addition, the increased number of FMM uptake regions significantly correlated with CSF biomarker levels toward a more AD-like pattern (increased Aß42 and decreased t-tau, t-tau/ Aß42). Our results are consistent with previous studies showing negative correlation between CSF Aß42 levels and PET-based quantitative uptake of ^18^F-florbetapir [31] or ^18^F-florbetaben [32].

Our third major finding was the order of Aß accumulation. Among the Focal-FMM-group, Aß deposition was most frequently observed in the frontal (62.3%) followed by the PPC (60.4%), parietal (60.4%), and least frequently observed in the striatum (43.4%) and lateral temporal (39.6%) regions. Unlike the Thal stage of amyloid deposition [9], our data showed that striatal involvement preceded the involvement of lateral temporal region. However, our result generally reflects a downward spreading pattern of Aß, suggesting that Aß deposits first in the cortex followed by subcortical structures [9]. Furthermore, our data revealed that, among Focal-FMM patients, those with subcortical Aß involvement (striatum) showed lower cognition than those with cortical Aß involvement (frontal, lateral temporal, parietal, and PPC). Our findings are concordant with those of previous studies which found that subcortical Aß involvement, especially striatal Aß, was related to worse cognitive performance and faster cognitive decline [33–35]. Patients with striatal involvement implies that they had higher Thal stage (Aß phase 3) and thus are more likely to have tau. Therefore, we suggest that even when Aß involvement is focal, Aß deposition in the striatum might be a sign of possible worse clinical outcome.

However, the present study has some limitations. First, our study used a cross-sectional design and, therefore, we do not know the cognitive trajectory of participants. Further longitudinal studies are warranted to evaluate the disease progression rate of the Focal-FMM group. Second, we lack pathological data on Focal-FMM patients. Amyloid PET-negative MCI or dementia patients in our data might have vascular, hippocampal sclerosis, or other pathologies as the main etiology for cognitive impairment. Further studies that could exclude non-AD pathologies are necessary. Nevertheless, the strength of our current study is that we have identified the clinical significance of focal Aß deposition, which comprised a substantial portion of participants in each cognitive level.

## Conclusions

In the current study, we found that focal Aß deposition has unique clinical characteristics that differ from patients with no or diffuse Aß deposition. We suggest that focal Aß deposition should be considered as an intermediate stage between no Aß and diffuse Aß deposition. In addition, when managing patients showing focal Aß deposition, clinicians should consider the number and location of regions of focal Aß deposition. Those with more regions involved, especially in the striatum, show clinical features close to diffuse Aß deposition. Thus, cognitively unimpaired or MCI individuals with such signs might be more closely monitored for future cognitive decline. Further longitudinal studies are needed to evaluate the disease progression of patients with focal Aß deposition.

## Supplementary information

Additional file 1: **Table S1.** Cognitive profiles according to the number of 18F-flutemetamol uptake regions. **Table S2.** Cognitive profile of No-, Focal-, and Diffuse-FMM groups in each cognitive level. **Table S3.** Number of uptake regions in cognitively unimpaired, mild cognitive impairment, and Alzheimer’s disease dementia. **Figure S1.** Examples of ^18^F-flutemetamol (FMM) PET scan in ‘No-FMM’, ‘Focal-FMM’ and ‘Diffuse-FMM’ groups. Red arrows and dashed circles show FMM uptake, while white arrows and dashed circles indicate no FMM uptake. (DOCX 2813 kb)

## Abbreviations

AD: Alzheimer’s disease
ADD: AD dementia
Aß: ß-amyloid
ANOVA: Analysis of variance
ANCOVA: Analysis of covariance
a-QC: Aqueous quality controls
CERAD-K: The Korean version of the Consortium to Establish a Registry for Alzheimer’s Disease Assessment Packet
COWAT: Controlled Oral Word Association Test
CU: Cognitively unimpaired
CDR-SB: Clinical Dementia Rating Scale Sum of Boxes
DSM-IV-TR: Diagnostic and Statistical Manual of Mental Disorders 4th Edition, Text Revision
FMM: ^18^F-Flutemetamol
KBASE-V: Korean Brain Aging Study for the Early Diagnosis and Prediction of AD
K-BNT: Korean version-Boston Naming Test
MCI: Mild cognitive impairment
MMSE: Mini-Mental State Examination
PPC: Precuneus/posterior cingulate
p-tau: Phosphorylated tau
RCFT: Rey-Osterrieth Complex Figure Test
SUVR: Standardized uptake value ratios
SNSB: Seoul Neuropsychological Screening Battery
t-tau: Total tau
