# Supplementary material for: Clinical significance of focal ß-amyloid deposition measured by 18F-flutemetamol PET
Source: Alzheimers Res Ther. 2020 Jan 4;12:6. doi: 10.1186/s13195-019-0577-x (PMC6942396; doi:10.1186/s13195-019-0577-x)
Supplement: Supplementary file 1 — Table S1. Cognitive profiles according to the number of 18F-flutemetamol uptake regions. Table S2. Cognitive profile of No-, Focal-, and Diffuse-FMM groups in each cognitive level. Table S3. Number of uptake regions in cognitively unimpaired, mild cognitive impairment, and Alzheimer’s disease dementia. Figure S1. Examples of 18F-flutemetamol (FMM) PET scan in ‘No-FMM’, ‘Focal-FMM’ and ‘Diffuse-FMM’ groups. Red arrows and dashed circles show FMM uptake, while white arrows and dashed circles indicate no FMM uptake. (DOCX 2813 kb) [file 13195_2019_577_MOESM1_ESM.docx]

**Table S1**. Cognitive profiles according to the number of 18F-flutemetamol uptake regions

|  | **No FMM uptake** | **Focal FMM uptake** | | **Diffuse FMM uptake** |
| --- | --- | --- | --- | --- |
|  | **(0 region)** | **(1-5 regions)** | **(6-9 regions)** | **(10 regions)** |
|  | **(n=174)** | **(n=35)** | **(n=18)** | **(n=83)** |
| **Language** |  |  |  |  |
| K-BNT | 0.02±1.17 | -0.51±1.48 | **-1.78±2.03*** | -0.97±1.50 |
| **Visuospatial** |  |  |  |  |
| Figure copying | -0.12±1.31 | **-0.01±1.14^#^** | **-2.08±3.00*** | -1.62±3.76 |
| **Memory** |  |  |  |  |
| Verbal memory | -0.46±1.26 | **-0.72±1.20^#^** | **-1.47±1.26*** | -2.12±1.04 |
| Visual memory | -0.18±1.10 | **-0.53±1.11^#^** | **-1.39±1.03*** | -1.77±0.96 |
| **Frontal/Executive** |  |  |  |  |
| COWAT | -0.07±1.10 | **-0.48±0.99^#^** | **-1.38±1.12*** | -1.09±1.11 |
| Stroop test | -0.06±1.42 | **-0.17±1.41^#^** | **-1.46±1.71*** | -1.66±1.77 |
| **Global** |  |  |  |  |
| MMSE | -0.31±1.35 | **-0.45±1.42^#^** | **-2.45±3.42*** | -2.57±2.98 |
| **CDR-SB** | 0.80±1.04 | **1.26±1.72^#^** | **3.94±3.17*** | 3.10±2.68 |

Values are mean z-scores of neuropsychological tests and raw score of CDR-SB (Mean±SD).

*p<0.017 after Bonferroni correction between regional Focal-FMM-uptake group and No-FM M-uptake group

^#^p<0.017 after Bonferroni correction between regional Focal-FMM-uptake group and Diffuse-FMM-uptake group

Abbreviations: FMM = ^18^F-flutemetamol; COWAT = The Controlled Oral Word Association Test; K-BNT = Korean version-Boston Naming Test; MMSE = Mini-Mental State Exam; CDR-SB = Clinical Dementia Rating Scale Sum of Boxes

**Table S2.** Cognitive profile of No-, Focal-, and Diffuse-FMM groups in each cognitive level

|  | **Cognitively unimpaired  (n=125)** | | |  | **Mild cognitive impairment**  **(n=125)** | | |  | **Alzheimer’s disease dementia**  **(n=60)** | | | |
| --- | --- | --- | --- | --- | --- | --- | --- | --- | --- | --- | --- | --- |
| **Neuropsychological tests of cognitive domain** | **No FMM uptake (n=102)** | **Focal FMM uptake (n=17)** | **Diffuse FMM uptake**  **(n=6)** |  | **No FMM uptake (n=64)** | **Focal FMM uptake (n=20)** | **Diffuse FMM uptake**  **(n=41)** |  | **No FMM uptake (n=8)** | **Focal FMM uptake (n=16)** | **Diffuse FMM uptake**  **(n=36)** |  |
| **Language** |  |  |  |  |  |  |  |  |  |  |  |  |
| K-BNT | 0.42±0.91 | 0.46±0.68 | 0.13±0.67 |  | -0.58±1.29 | -0.81±1.05 | -0.71±1.54 |  | -0.22±1.22 | **-2.59±1.98*** | -1.45±1.40 |  |
| **Visuospatial** |  |  |  |  |  |  |  |  |  |  |  |  |
| Figure copying | 0.24±0.63 | 0.38±0.62 | -0.14±1.05 |  | -0.62±1.75 | -0.31±1.85 | -1.00±2.13 |  | -0.66±2.13 | -2.38±2.70 | -2.56±5.10 |  |
| **Memory** |  |  |  |  |  |  |  |  |  |  |  |  |
| Verbal memory | 0.26±0.86 | 0.27±0.97 | -0.35±0.62 |  | -1.42±1.02 | **-1.26±0.97^#^** | -2.04±0.97 |  | -1.95±0.47 | **-1.94±0.65^#^** | -2.50±0.83 |  |
| Visual memory | 0.36±0.89 | 0.16±0.99 | -0.81±0.81 |  | -0.89±0.95 | **-0.99±1.02^#^** | -1.64±0.97 |  | -1.32±0.46 | -1.68±0.55 | -2.08±0.84 |  |
| **Frontal/Executive** |  |  |  |  |  |  |  |  |  |  |  |  |
| COWAT | 0.26±1.06 | -0.01±1.09 | -0.57±1.41 |  | -0.49±1.04 | -0.62±0.60 | -0.83±1.10 |  | -0.80±0.50 | **-1.82±0.85*** | -1.48±0.98 |  |
| Stroop test | 0.41±1.30 | 0.57±1.08 | -0.54±0.39 |  | -0.61±1.34 | **-0.33±1.11^#^** | -1.33±1.59 |  | -1.55±0.95 | -2.19±1.43 | -2.22±1.94 |  |
| **Global** |  |  |  |  |  |  |  |  |  |  |  |  |
| MMSE | 0.29±0.85 | 0.38±0.81 | -0.16±0.57 |  | -0.99±1.39 | **-0.54±1.06^#^** | -1.67±2.04 |  | -2.44±1.66 | -3.48±3.15 | -4.01±3.46 |  |
| **CDR SB** | 0.26±0.42 | 0.18±0.35 | 0.20±0.45 |  | 1.36±0.82 | 1.30±0.77 | 1.51±0.93 |  | 3.13±2.20 | 5.67±2.30 | 5.32±2.52 |  |

Values are mean z-scores of neuropsychological tests and raw score of CDR-SB (Mean±SD).

*p<0.017 after Bonferroni correction between Focal-FMM-uptake group and No-FMM-uptake group

#p<0.017 after Bonferroni correction between Focal-FMM-uptake group and Diffuse-FMM-uptake group

Abbreviations: CU = cognitively unimpaired; MCI = mild cognitive impairment; ADD = Alzheimer’s disease dementia; FMM = ^18^F-flutemetamol; PPC = precuneus/posterior cingulate; COWAT = The Controlled Oral Word Association Test; K-BNT = Korean version-Boston Naming Test; MMSE = Mini-Mental State Exam; CDR-SB = Clinical Dementia Rating Scale Sum of Boxes

**Table S3.** Number of uptake regions in cognitively unimpaired, mild cognitive impairment, and Alzheimer's disease dementia

| **No. of Aβ uptake regions** | **CU (n=125)** | **MCI (n=125)** | **ADD (n=60)** |
| --- | --- | --- | --- |
| **No-FMM uptake** |  |  |  |
| 0 region | 102 (81.6%)^*^ | 64 (51.2%)† | 8 (13.3%)^‡^ |
| **Focal-FMM uptake** |  |  |  |
| 1 region | 9 (7.2%) | 4 (3.2%) | 0 (0.0%) |
| 2 regions | 2 (1.6%) | 4 (3.2%) | 2 (3.3%) |
| 3 regions | 0 (0.0%) | 2 (1.6%) | 2 (3.3%) |
| 4 regions | 3 (2.4%) | 3 (2.4%) | 2 (3.3%) |
| 5 regions | 1 (0.8%) | 1 (0.8%) | 0 (0.0%) |
| 6 regions | 0 (0.0%) | 2 (1.6%) | 3 (5.0%) |
| 7 regions | 0 (0.0%) | 2 (1.6%) | 2 (3.3%) |
| 8 regions | 2 (1.6%) | 2 (1.6%) | 4 (6.7%) |
| 9 regions | 0 (0.0%) | 0 (0.0%) | 1 (1.7%) |
| **Diffuse-FMM uptake** |  |  |  |
| 10 regions | 6 (4.8%)^*^ | 41 (32.8%)† | 36 (60.0%)^‡^ |

*p<0.017 after Bonferroni correction between CU and MCI

†p<0.017 after Bonferroni correction between MCI and ADD

^‡^p<0.017 after Bonferroni correction between ADD and CU

Abbreviations: FMM = ^18^F-flutemetamol; CU = cognitively unimpaired; MCI = mild cognitive impairment; ADD = Alzheimer’s disease dementia

**Figure Legend**


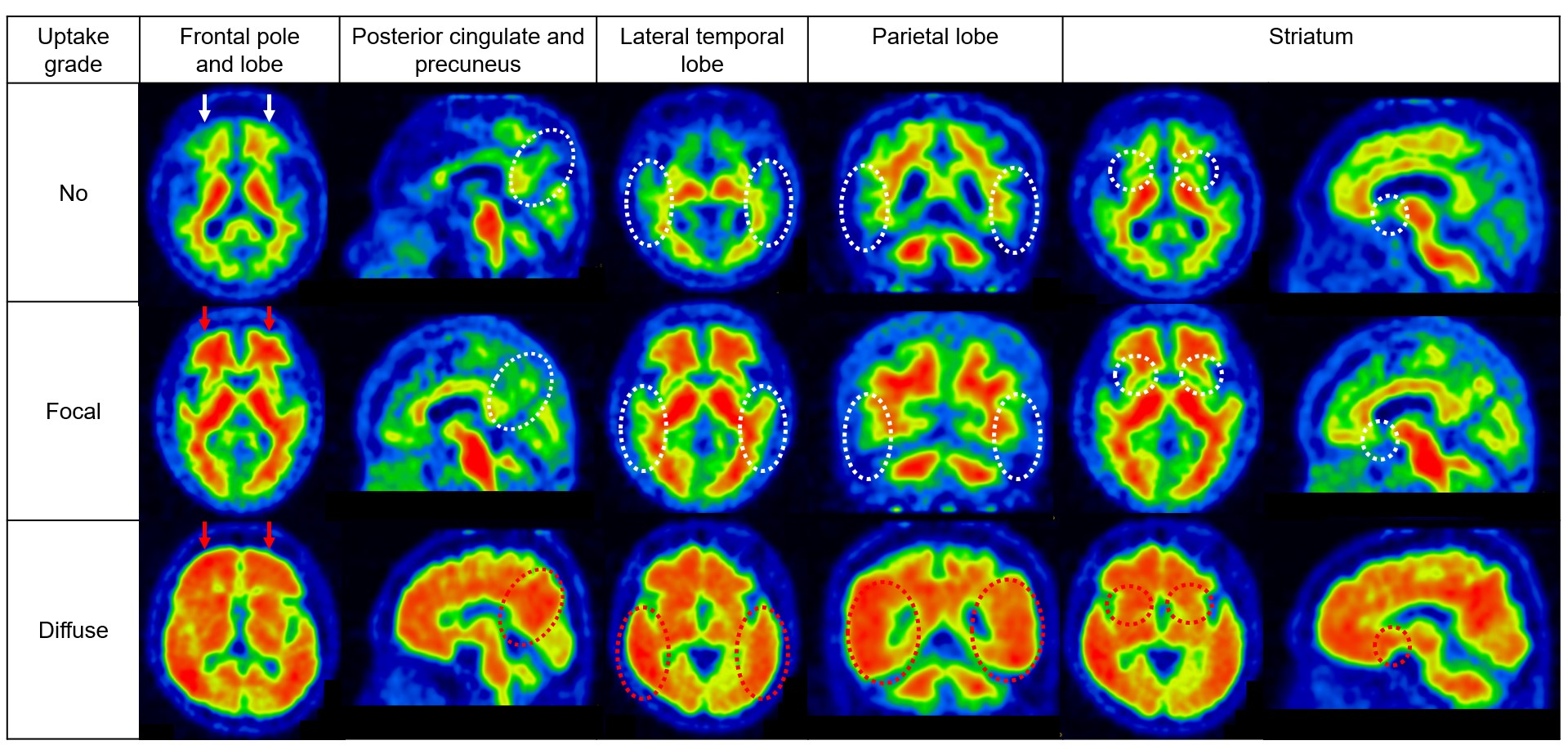


**Fig. S1.** Examples of ^18^F-flutemetamol (FMM) PET scan in ‘No-FMM’, ‘Focal-FMM’ and ‘Diffuse-FMM’ groups. Red arrows and dashed circles show FMM uptake, while white arrows and dashed circles indicate no FMM uptake.
